# Infiltrating regulatory T cell numbers is not a factor to predict patient's survival in oesophageal squamous cell carcinoma

**DOI:** 10.1038/sj.bjc.6604294

**Published:** 2008-03-18

**Authors:** T Yoshioka, M Miyamoto, Y Cho, K Ishikawa, T Tsuchikawa, M Kadoya, L Li, R Mishra, K Ichinokawa, Y Shoji, Y Matsumura, T Shichinohe, S Hirano, T Shinohara, T Itoh, S Kondo

**Affiliations:** 1 Department of Surgical Oncology, Division of Cancer Medicine, Hokkaido University Graduate School of Medicine, N-15, W-7, Sapporo, Hokkaido 060-8638, Japan; 2 Department of pathology, Teine Keijinkai Hospital, Hokkaido 060-8638, Japan; 3 Department of pathology, Hokkaido University Hospital, Hokkaido 060-8648, Japan

**Keywords:** OSCC, Foxp3, T_reg_, anti-tumour immunity, prognosis

## Abstract

CD4/8 status has been previously reported to be a critical factor in the prognosis of oesophageal squamous cell carcinoma (OSCC). In the current study, we investigated the effect of regulatory T cells (T_reg_; Foxp3^+^ lymphocytes) on the status of CD4^+^ and CD8^+^ T cells in 122 patients with OSCC. Immunohistochemical analysis of T_reg_ was performed using an antibody against Foxp3. The survival rate for low Foxp3 patients was significantly lower than for high Foxp3 patients (*P*=0.0028 by log-rank test), but Foxp3 status did not significantly correlate with prognosis in CD4/8(+/+) patients or CD4/8(+/−) or (−/+) patients (*P*=0.5185 and 0.8479, respectively, by log-rank test). We also found that Foxp3 status correlated with CD4/8 status (*P*=0.0002 by *χ*^2^ test) and that the variance of CD8/CD4 ratio in patients with low Foxp3 was larger than in patients with high Foxp3 (*P*<0.0001 by F-test). Thus, the results do not support the idea that T_reg_ suppress anti-tumour immunity in patients with OSCC. Rather, the CD8/CD4 ratio and CD4/8 status appear to be critical factors in anti-tumour immunity. Furthermore, T_reg_ numbers correlate with both the CD8/CD4 ratio and the CD4/8 status, suggesting that T_reg_ number is not a factor to predict patient's survival in OSCC.

In previous studies, we found that cooperation between CD4^+^ and CD8^+^ T cells appears to drastically improve the prognosis of patients with oesophageal squamous cell carcinoma (OSCC) (Cho *et al*, 2003). Thus, the host immune response against cancer cells appears to play a critical role in the inhibition of recurrence and determines the postsurgical prognosis in OSCC. Recent reports suggested that thymic-derived CD4^+^CD25^+^ regulatory T cells (T_reg_; Foxp3^+^ lymphocytes) participate in the control of tumour immunity, but whether T_reg_ control tumour immunity in OSCC has not been established.

T_reg_ represent a minor fraction (5–10%) of the CD4^+^ T cells, and they maintain immune homoeostasis in immunotolerance and control autoreactive T cells. In addition to their role in autoimmunity, T_reg_ participate in transplantation tolerance and tumour immunity. Although the mechanisms of suppression by T_reg_ remain to be determined *in vivo*, many investigators have reported that T_reg_ can inhibit immune responses mediated by CD4^+^CD25^−^ T cells and CD8^+^ T cells *in vitro* via cell–cell contact (Sakaguchi *et al*, 1995; Sakaguchi, 2000; Shevach, 2002; Camara *et al*, 2003; Anatomy *et al*, 2005). T_reg_ express CD25 (interleukin-2 receptor *α*), glucocorticoid-induced tumour necrosis factor receptor, and cytolytic T lymphocyte-associated antigen 4 on their surface. The nuclei of these cells also contain Foxp3, which is a member of the forkhead or winged helix family of transcription factors. Foxp3 is reported to be a key regulatory gene for the development and function of T_reg_ and the most specific marker of T_reg_ (Chatila *et al*, 2000; Brunkow *et al*, 2001; Mchugh *et al*, 2002; Fontenot *et al*, 2003; Hori *et al*, 2003; Khattri *et al*, 2003; Fontenot and Rudensky, 2005).

Several studies in mice have shown that T_reg_ inhibit the anti-tumour immune response (Onizuka *et al*, 1999; Shimizu *et al*, 1999; Nishikawa *et al*, 2005) and that depletion of T_reg_ can enhance effector T cell anti-tumour responses (Sutmuller *et al*, 2001; Tanaka *et al*, 2002). Additional studies have reported that the T_reg_ population increases in peripheral blood and tumour tissues from patients with several types of human cancer (Woo *et al*, 2001; Liyanage *et al*, 2002; Ichihara *et al*, 2003; Curiel *et al*, 2004; Kawaida *et al*, 2005; Ormandy *et al*, 2005), but the relationship between the T_reg_ population and the prognosis has not been clarified in OSCC. The purpose of this study, therefore, was to determine the effect of T_reg_ on CD4^+^ and CD8^+^ T cells in OSCC. The present study was performed with same cohort as in Cho *et al* (2003).

## MATERIALS AND METHODS

### Patients and specimens

One hundred and twenty-two patients (105 male and 17 female; mean age, 62.3 years) with primary OSCC underwent radical oesophagectomy between September 1989 and May 1999 at the Department of Surgical Oncology, School of Medicine, Hokkaido University or at an affiliated hospital (Department of Surgery, Teine keijinkai Hospital and Department of Surgery, Hokkaido Gastroenterology Hospital). Preoperative examination did not find distant metastasis in any of the patients, and none of the patients had received prior anticancer treatments. Cases of in-hospital death were excluded from the current study. The clinical typing of tumours was determined according to the tumour-node-metastasis (TNM) classification system of the International Union Against Cancer (Sobin and Wittekind, 2002). All tumour specimens were fixed in 10% formalin and embedded in paraffin wax. One of the deepest sections from each tumour was selected for evaluation, and serial 4-μm thick sections were examined by immunohistochemistry. All the informed consent process for immunohistochemical staining were conducted in accordance with the guidelines of the Hokkaido University Institutional Review Board Authorization for this study.

### Immunohistochemistry

For immunohistochemical analysis, formalin-fixed and paraffin-embedded specimens were deparaffinised in xylene and dehydrated through a graded series from ethanol to water. For antigen retrieval, sections were floated on 1 mM EDTA buffer (pH 9.0) in a plastic container and then heated in a domestic pressure cooker for 3 min after it reached the maximum pressure. Once cooled, the heat-treated sections were washed three times for 5 min each with PBS (pH 7.4). Before staining the sections, endogenous peroxidase activity was eliminated by a 20-min incubation in 0.3% hydrogen peroxide in methanol. After washing in PBS, specimens were blocked with 10% normal goat serum (Nichirei Corporation, Tokyo, Japan) for 30 min and then incubated at room temperature for 60 min with 1 : 40 mouse anti-human Foxp3 antibody (clone 246A/E7; Abcam, Cambridge, UK) in antibody diluent (DakoCytomation, Glostrup, Denmark). Normal adenoid tissue was used as a positive control for Foxp3. After washing with PBS, the sections were incubated for 60 min at room temperature with a biotinylated goat antibody to mouse immunoglobulin (Histofine Simple Stain MAX PO [MALTI]; Nichirei Corporation, Tokyo, Japan). After washing in PBS, immunohistochemical staining was developed by incubating the sections in freshly prepared 3,3′-diaminobenzidine tetrahydrochloride (Histofine Simple Stain DAB Solution; Nichirei Corporation) for approximately 10 min. The sections were washed in distilled water, counterstained with haematoxylin for 15 s, and mounted in Permount (Micro Slides; Muto-Glass, Tokyo, Japan). Mouse IgG1 (DakoCytomation, Glostrup, Denmark) was used in place of the Foxp3 antibody for negative controls.

### Quantification of T_reg_

Immunohistochemically stained sections were evaluated under a microscope (Olympus Optical Co. Ltd, Tokyo, Japan). The current study was performed in a retrospective manner, but all specimens were evaluated by two investigators blinded to the patients’ clinical information. T_reg_ were quantified by analyzing five different high power fields ( × 400). Between 0 and 848 T_reg_ were detected in the five fields, and the median (109) was used as a cutoff to define the subgroups.

### Statistical analysis

Statistical analysis was performed using the *χ*^2^ test. The Kaplan–Meier method was used to estimate overall survival and survival differences were analysed by the log-rank test based on the number of immune cells. Univariate and multivariate analyses of immune cells and clinicopathological features were performed using the Cox proportional hazard regression model. The F-test was used to analyse the variance in the CD8/CD4 ratio. The Mann–Whitney *U*-test was used to analyse the number of T_reg_. In all cases, *P*-values less than 0.05 were regarded as indicating statistical significance. All statistical analyses were performed using StatView J version 5.0 (SAS Institute Inc., Cary, NC, USA).

## RESULTS

### Immunohistochemical staining of T_reg_

**Figure 1:**
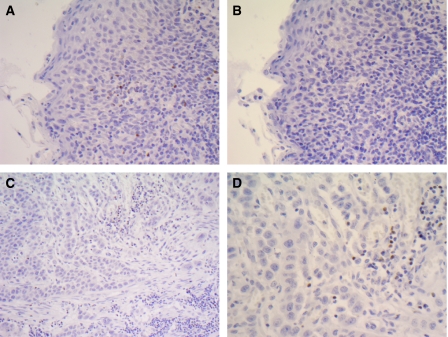
Immunohistochemical staining. (**A**) Positive control tonsil stained with anti-human Foxp3 antibody. (**B**) Negative control tonsil stained with isotype-matched IgG. (**C**, **D**) OSCC stained with anti-human Foxp3 antibody at (**C**) × 200 and (**D**) × 400.

Figure 1 shows representative photomicrographs of immunohistochemical staining for T_reg_ using an antibody to human Foxp3. T_reg_ were detected in cancer cell nests or in the stroma in contact with cancer cells.

### Correlation between Foxp3 status and clinicopathological features

Correlations between Foxp3 status and various clinicopathological features are summarised in Table 1. Foxp3 status was found to correlate with CD4 status (*P*=0.0186), CD8 status (*P*=0.0021), and CD4/8 status (*P*=0.0002). No significant correlation was found between Foxp3 status and age, gender, pathological data according to TNM classification, or p-stage grouping.

### Kaplan–Meier survival analysis of low Foxp3 and high Foxp3 patients

**Figure 2:**
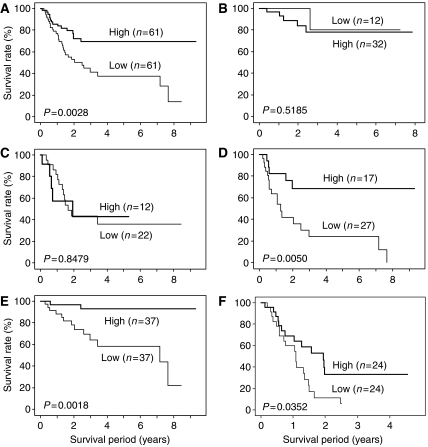
Kaplan–Meier analyses of overall survival according to the number of T_regs_ in (**A**) all patients (*n=122*), (**B**) CD4/8(+/+) patients (*n*=44), (**C**) CD4/8(+/−) and (−/+) patients (*n*=34), (**D**) CD4/8 (−/−) patients (*n*=44), (**E**) stage I and II patients (*n*=74), and (**F**) stage III and IV patients (*n*=48).

Survival curves were constructed according to the Kaplan–Meier method (Figure 2). In the 122 patients with OSCC (Figure 2A), the survival rates for patients with low Foxp3 were significantly lower than for patients with high Foxp3 (*P*=0.0028 by log-rank test). In CD4/8(+/+) patients (*n*=44; Figure 2B) or CD4/8 (+/−) or (−/+) patients (*n*=34; Figure 2C), Foxp3 status was not significantly related to the prognosis (*P*=0.5185 and 0.8479, respectively, by log-rank test). In CD4/8(−/−) patients (*n*=44; Figure 2D), the survival rates for patients with low Foxp3 were significantly lower than for patients with high Foxp3 (*P*=0.0050 by log-rank test). Similar results were found in patients divided into subgroups of p-stages I/II (*P*=0.0018; Figure 2E) and III/IV (*P*=0.0352; Figure 2F).

### Univariate and multivariate analyses

Univariate analysis for overall survival using a Cox regression model identified T classification, N classification, M classification, CD4 status, CD8 status, and Foxp3 status as significant predictors of the prognosis. Multivariate analysis of the same set of patients was performed for pathological predictors, CD4 status, CD8 status, and Foxp3 status for survival time using the Cox regression model. T classification, N classification, CD8 status, and Foxp3 status were of independent prognostic value (Table 2). Although Foxp3 status was not independent when multivariate analysis was performed using CD4/8 status and Foxp3 as factors, Foxp3 status had independent prognostic value in selected cases of CD4/8(−/−) patients (Hazard ratio=3.382; *P*=0.0207; data not shown).

### Correlation between CD4/8 status and the number of T_reg_

**Figure 3:**
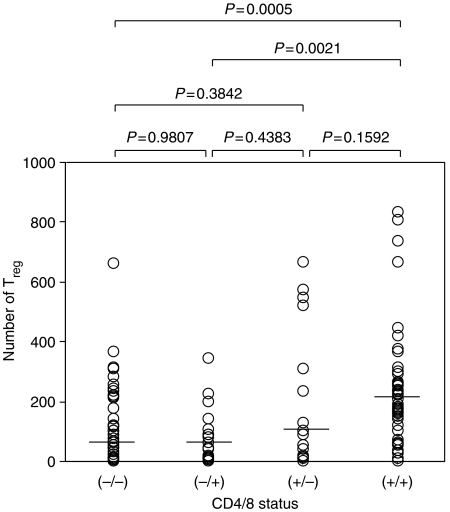
Correlation between CD4/8 status and the number of T_regs_. The median number of T_regs_ in CD4/8(−/−), CD4/8(−/+), CD4/8(+/−), and CD4/8(+/+) patients was 67, 68, 93 and 203, respectively.

A significant correlation was found between CD4/8 status and the number of T_reg_ by the Mann–Whitney *U*-test (for CD4/8(+/+) *vs* (−/+), *P*=0.0021; for CD4/8 (+/+) *vs* CD4/8 (−/−), *P*=0.0005; Figure 3). The median value of the number of T_reg_ in CD4/8(+/+), CD4/8(+/−), CD4/8(−/+), and CD4/8(−/−) patients was 203, 93, 68 and 67 respectively.

### Correlation between Foxp3 status and the variance on CD8/CD4 ratio

**Figure 4:**
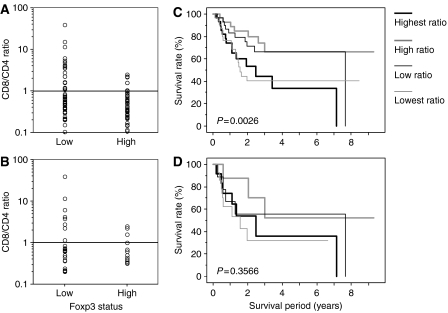
Correlation between the variance in the CD8/CD4 ratio and Foxp3 status (**A**, **B**) and Kaplan–Meier analyses of overall survival relative to the CD8/CD4 ratio (**C**, **D**) in all patients with OSCC (**A**, **C**) and in CD4/8(−/−) patients (**B**, **D**).

In the 122 patients with OSCC (Figure 4A), the heterogeneity of the variance in the CD8/CD4 ratio was significantly different between high Foxp3 patients and low Foxp3 patients (*P*<0.0001 by F-test). The variance on CD8/CD4 ratio of low Foxp3 patients (variance: 30.046) was larger than high Foxp3 patients (variance: 0.279) in the 122 patients with OSCC. In selected case of CD4/8(−/−), similarly, the variance on CD8/CD4 ratio was 58.073 in low Foxp3 patients and 0.766 in high Foxp3 patients (Figure 4B).

### Kaplan–Meier survival analysis according to the CD8/CD4 ratio

The patients were divided into four groups according to their CD8/CD4 ratios (Figure 4C). The survival rates for patients with the highest and lowest CD8/CD4 ratios were significantly lower than for the other two groups (*P*=0.0026 by log-rank test). In the CD4/8(−/−) patients (*n*=44; Figure 4D), the CD8/CD4 ratio was not significantly related to the prognosis (*P*=0.3566 by log-rank test).

## DISCUSSION

In recent years, factors that regulate immune responses to malignant tumour cells have received a great deal of attention. Several of these studies have shown that T_reg_ are recruited to human carcinomas and they may influence the prognosis of cancer patients (Woo *et al*, 2001; Liyanage *et al*, 2002; Ichihara *et al*, 2003; Curiel *et al*, 2004; Kawaida *et al*, 2005; Ormandy *et al*, 2005). To adequately evaluate antitumour immune function; however, multiple factors should be evaluated simultaneously. Here, we found that the CD8^+^ T cell effect was significant only when CD4^+^ T cells were also present. This is the first report that has clarified the correlation of T_reg_ with CD4^+^ and CD8^+^ T cells in OSCC and examined the effect of T_reg_ on these T cells. In addition, we did not find a correlation between Foxp3 status and TNM classification, suggesting that T_reg_ do not influence the progression of cancer.

Based on evidence from murine models, the prognosis of high Foxp3 patients is expected to be poor (Onizuka *et al*, 1999; Shimizu *et al*, 1999; Sutmuller *et al*, 2001; Tanaka *et al*, 2002; Nishikawa *et al*, 2005); however the current results do not support this hypothesis because the high Foxp3 patients had a dramatically better prognosis than the low Foxp3 patients. Thus, the increase in T_reg_ seemed to be due to an increase in the total number of T lymphocytes, and T_reg_ do not appear to suppress the anti-tumour immune response. In fact, the number of T_reg_ in CD4/8(+/+) patients was significantly higher than in CD4/8(−/−) patients and CD4/8(−/+) patients, and survival curves divided on the basis of Foxp3 status in CD4/8(+/+) patients were similar. Interestingly, in CD4/8(−/−) patients, the survival rates for low Foxp3 patients were significantly lower than for high Foxp3 patients. Given these results, it appears that the presence of T_reg_ suggested a normal antitumour immune response, and T_reg_ do not appear to inhibit the proliferation of tumour-specific T lymphocytes. Similar results were shown in patients divided on the basis of p-stage. Curiel *et al* (2004) reported that tumour cells produce the chemokine CCL22, which mediates trafficking of T_reg_ to the tumour and that the percentage of T_reg_ in CD4^+^CD3^+^ T cells is higher in the later than the earlier stage of disease; however, we did not find a correlation between p-stage and Foxp3 status, and high Foxp3 patients had a better prognosis in both earlier stage and advanced stage patients. Although the intratumor balance of T_reg_ and CD8^+^ T cell has been shown to correlate with prognosis of several cancer types such as ovarian cancer and hepatocellular carcinoma (Sato *et al* 2005; Gao *et al* 2007), we did not find a significant correlation between CD8^+^ T cell/T_reg_ ratio and prognosis. These data also support the idea that T_reg_ do not suppress antitumour immunity in OSCC.

Various immunological factors must be considered to understand the state of antitumour immunity. We previously reported that the prognosis of the CD4/8(+/+) group is remarkably better than that of the other groups (Cho *et al*, 2003). Around the same time, Diederichsen Axel *et al* (2003) reported that a high CD8/CD4 ratio is associated with a better prognosis in colorectal cancer. Furthermore, Piersma *et al* (2007)also reported that a higher CD8/CD4 ratio is associated with the lack of tumour metastases in the draining lymph nodes of cervical cancer patients. Therefore, it is obvious that the balance of CD4^+^ T cell and CD8^+^ T cell is critical for prognosis of patients with cancer. In the present study, the favourable prognosis of high Foxp3 patients in the CD4/8(−/−) group appears to be related to the CD8/CD4 ratio. When the patients were divided into four groups on the basis of the CD8/CD4 ratio, the prognoses of the groups with the highest and lowest ratios were poorer than those of the other two groups. In addition, the CD8/CD4 ratio was disrupted in low Foxp3 patients with poorer prognoses. Similar results were observed in the CD4/8(−/−) patients. These findings show that a suitable CD4/8 ratio corresponds with a favourable prognosis and that it reflects the immune response against OSCC.

In conclusion, the increase of infiltrating T_reg_ in OSCC patients is due to an increase in the total number of T lymphocytes, and the results did not support the idea that T_reg_ suppress anti-tumour immunity in patients with OSCC. Rather, the CD8/CD4 ratio and CD4/8 status appear to be critical for anti-tumour immunity, and infiltrating T_reg_ correlates with both of these measures of anti-tumour immune function in OSCC.

## Figures and Tables

**Table 1:** Correlation between clinicopathological features of the 122 patients with OSCC and the number of Foxp3 positive cells

		**Foxp3 positive cell**		
		**Low**	**High**	
**Variable**	**No. of cases**	***n*=61**	***n*=61**	***P*-value**
*Age (Years)*				
<62	58	28	30	0.7169
⩾62	64	33	31	
				
*Gender*				
Male	105	53	52	0.7938
Female	17	8	9	
				
*pT classification*				
T1/T2	67	32	35	0.5852
T3/T4	55	29	26	
				
*pN classification*				
Negative	60	28	32	0.4688
Positive	62	33	29	
				
*pM classification*				
M0	101	50	51	0.8105
M1	21	11	10	
				
*P-Stage*				
I/II	76	38	38	>0.9999
III/IVA	46	23	23	
				
*CD4 status*				
Abundant	61	24	37	0.0186
Scanty	61	37	24	
				
*CD8 status*				
Abundant	61	22	39	0.0021
Scanty	61	39	22	
				
*CD4/8 status*				
CD4/8(+/+)	44	12	32	0.0002
Others	78	49	29	

**Table 2:** Univariate and multivariate analyses of immune cells and clinicopathological features using the Cox proportional hazard regression model

	**Univariate**		**Multivariate**	
**Variable**	**Hazard ratio (95% CI)**	***P*-value**	**Hazard ratio (95% CI)**	***P*-value**
Gender (male/female)	2.812 (0.872–9.070)	0.0835		
Age (⩾62 *vs* <62) (years)	1.404 (0.783–2.519)	0.2543		
pT classification (3/4 *vs* 1/2)	4.164 (2.238–7.747)	<0.0001	2.564 (1.305–5.038)	0.0063
pN classification (1 *vs* 0)	5.880 (2.885–11.985)	<0.0001	5.051 (2.273–11.236)	<0.0001
pM classification (1 *vs* 0)	3.056 (1.615–5.780)	0.0006	1.067 (0.532–2.141)	0.8556
pStage (III/IV *vs* I/II)	7.828 (3.969–15.437)	<0.0001		
CD4 status (abundant *vs* scanty)	0.350 (0.184–0.664)	0.0013	0.778 (0.374–1.619)	0.5022
CD8 status (abundant *vs* scanty)	0.451 (0.248–0.819)	0.0089	0.502 (0.259–0.974)	0.0417
Foxp3 status (low *vs* high)	2.474 (1.338–4.577)	0.0039	2.239 (1.172–4.275)	0.0146
				
CD4/8 (CD4/8(+/+) *vs* others)	0.208 (0.088–0.492)	0.0003	0.250 (0.130–0.603)	0.0020
Foxp3 status (low *vs* high)	2.474 (1.338–4.577)	0.0039	1.784 (0.949–3.353)	0.0722

CI, confidence interval.
